# Pannexin-1–mediated ATP signaling as a driver of immune communication and chronic inflammation in HIV infection

**DOI:** 10.3389/fimmu.2026.1836368

**Published:** 2026-04-30

**Authors:** Emma K. Kaufman, Talia H. Swartz

**Affiliations:** Division of Infectious Diseases, Department of Medicine, Immunology Institute, Icahn School of Medicine at Mount Sinai, New York, NY, United States

**Keywords:** ATP, HIV, NLRP3 inflammasome, pannexin channels, purinergic signaling, P2X7

## Abstract

Chronic inflammation is a hallmark of many diseases, including persistent viral infections. In human immunodeficiency virus (HIV) infection, sustained immune activation contributes to end-organ damage and non-AIDS comorbidities, even during antiretroviral therapy. This mini-review examines the role of Pannexin-1 (PANX1) channel–mediated ATP signaling as a key mechanism of immune communication driving chronic inflammation in HIV infection. Viral engagement of host receptors, such as CD4 and CXCR4, induces PANX1 channel opening in infected or stressed cells, leading to the release of extracellular ATP. Extracellular ATP acts as a danger-associated molecular pattern (DAMP) that engages purinergic receptors. Activation of P2X7 initiates potassium efflux, NLRP3 inflammasome assembly, and caspase-1 cleavage, leading to the maturation of the pro-inflammatory cytokines IL-1β and IL-18. The review integrates findings from cellular, molecular, and neuroimmunologic studies to highlight how this PANX1–ATP–P2X7 axis amplifies intercellular communication and propagates chronic immune activation. By positioning HIV as a model system, we discuss how insights from this pathway may extend to other chronic inflammatory conditions. Finally, we explore therapeutic opportunities for targeting PANX1 channels or purinergic receptors to reduce inflammasome-driven pathology. Understanding the mechanisms by which extracellular ATP coordinates immune cell signaling provides a framework for decoding chronic inflammation and designing precision interventions to restore immune balance.

## Introduction

Human Immunodeficiency Virus Type 1 (HIV-1) remains a global challenge, affecting over 40 million people (1). Progress in the development of antiretroviral therapy (ART) combined with early diagnosis and consistent treatment has made it possible for people with HIV (PWH) to live within a few years of the average life expectancy (2). However, even with an undetectable viral load, episodic background viral reactivation drives persistent immune activation associated with end-organ damage, cardiovascular disease, and a higher prevalence of age-associated comorbidities occurring at a younger age (3, 4). Early establishment of infection in the central nervous system (CNS) combined with the limited penetration of some antiretroviral drugs across the blood–brain barrier allows glial cells to act as a reservoir for both integrated latent provirus and replication-competent virus (5–7). Resulting sustained CNS inflammation is thought to contribute to the development of HIV-associated neurocognitive disorders (HAND), experienced by a substantial proportion of PWH, with symptoms ranging from memory issues to difficulty with coordination and walking (8, 9). Identifying host mechanisms coupling HIV infection with inflammatory signaling could provide insight into strategies to disrupt the pathway and reduce subsequent inflammation.

The NLRP3 inflammasome is a well-characterized protein complex integral to innate immunity through the release of the pro-inflammatory cytokines IL-18 and IL-1β (10). Inflammasome activation occurs in a two-step model: priming by NF-kB-dependent upregulation of NLRP3 genes and activation by a second signal that causes the cleavage of caspase-1 and the subsequent processing and release of biologically active IL-18 and IL-1β (11–13). One of many redundant inflammasome activation pathways, the PANX1-ATP-P2X7 axis acts as a potent amplifier and context-dependent regulator of inflammation.

Pannexin-1 (PANX1) channels are ubiquitous transmembrane hemichannels, key mediators of intercellular signaling through regulated release of extracellular ATP (14). Released at the initiation of apoptosis, ATP acts as a “find me” signal to attract phagocytes to dispose of cell products after apoptosis (15). A sign of cellular distress, extracellular ATP signals to neighboring cells via purinergic receptors, particularly P2X7. This interaction acts as a second signal for NLRP3 activation, driving ion flux, including potassium efflux and calcium signaling, which promote caspase-1 activation (16–18). PANX1 is also implicated in the propagation of calcium waves, or ATP-induced ATP release. Extracellular ATP binds to purinergic P2Y receptors, either on the cell from which it was released or a neighboring cell, initiating the release of calcium into the cytoplasm. The increase in cytoplasmic calcium triggers the opening of PANX1 channels and release of ATP (19, 20). In the setting of persistent stimuli such as HIV infection, this establishes a sustained ATP-dependent signaling loop that amplifies inflammatory responses.

Collectively, these findings define a model in which the PANX1–ATP–P2X7 axis functions as a feed-forward signaling circuit linking viral entry, intercellular communication, and inflammasome activation. Within this framework, ATP release is not simply a downstream consequence of infection, but an active mediator of both viral propagation and bystander immune activation. This review examines this axis as a unified signaling module with dual roles in HIV pathogenesis: facilitating viral spread and sustaining chronic immune activation. We further explore therapeutic opportunities to attenuate NLRP3 inflammasome activation by inhibiting pannexin channels and purinergic receptors. Key unanswered questions remain regarding this pathway as a potential therapeutic target, including long-term safety and the identification of patient populations most likely to benefit from modulation of ATP-mediated signaling as an adjuvant to ART. Although chronic inflammation is not unique to PWH, the framework developed through the study of immune signaling in HIV-1 may inform the design of interventions to mitigate the clinical consequences of chronic inflammation across diseases.

## The proviral arm of the PANX1–ATP–P2X7 axis: facilitation of viral transmission

HIV-1 can be transmitted through cell-free or cell-cell contact via the virologic synapse (21), which enables more efficient viral spread. PANX1 has been found to be associated with these virological synapses (22). Early in infection, the binding of HIV envelope protein gp120 to host CD4 and viral co-receptors CXCR4/CCR5 stimulates the intermittent opening of PANX1 channels (22–24). Extracellular ATP engages P2Y2 receptors to trigger depolarization of the plasma membrane, stimulating fusion, and the formation of the viral synapse (22). In later stages of infection, with both X4 and R5 HIV isolates, PANX1 channels remain open persistently, creating a feedback loop that facilitates infection (23–25). Extracellular ATP released can also propagate viral spread through engaging with the purinergic receptor P2X7 to induce the release of virions from infected macrophages (26).

## The immunopathogenic arm: amplification of inflammation in neurohiv

ATP signaling in the CNS involves PANX1 channels and P2X7 receptors. Circulating ATP has been identified as a predictive biomarker of cognitive impairment in PWH (25, 27). While the blood-brain barrier (BBB) usually regulates diffusion between blood and the CNS, HIV infection damages this barrier, increasing permeability to circulating ATP (28). High levels of circulating ATP activate the NLRP3 inflammasome via P2X7, leading to the release of the pro-inflammatory cytokine IL-1β and subsequent downstream disruption of the tight junction proteins in the epithelial cells that make up the BBB (18, 29). Elevated extracellular ATP within the CNS because of this P2X7 receptor-dependent ATP-induced BBB permeability can engage with purinergic receptors on astrocytes and neuronal cells, amplifying the inflammatory response (24, 30).

Simian immunodeficiency virus (SIV) infection in macaques, a well-established model to mimic the effects of HIV, has been identified as a cause of PANX1-dependent synaptic compromise in a manner associated with disruption of the BBB (28). This damage to the CNS in addition to ATP-induced BBB permeability suggests the reduction of circulatory ATP as an upstream target to prevent not only general chronic inflammation, but also to address HAND.

## Therapeutic opportunities

### Pannexin channel inhibition

Inhibition of PANX1 has been shown to reduce HIV-induced cell death, inhibit infection, and prevent fusion between infected cells and viral co-receptors CD4/CCR5 (22). Small interference RNA (siRNA) is used in the investigation of specific protein functions, such as PANX1, to precisely downregulate protein expression. siRNA targeting PANX1 was integral in the discovery of the upstream role of PANX1 in the P2X7-mediated NLRP3 inflammasome pathway, blocking caspase-1 cleavage and the subsequent release of IL-1β in macrophages (18). Orellana et al. demonstrated the role of PANX1 in HIV-induced pore formation utilizing siRNA. By knocking down PANX1 in PBMCs and CD4+ T lymphocytes and observing the corresponding decrease in dye uptake upon HIV infection, it was possible to conclude that inhibition of PANX1 could be a method of preventing ATP release during HIV infection (23). This conclusion was confirmed by Séror et al. (22).

The mimetic peptide 10Panx similarly inhibits PANX1 opening. Macrophages treated with 10Panx behave similarly to those with siRNA targeting PANX1 (18). In their macaque study, Gorska et al. proposed that inhibition of PANX1 with the mimetic peptide 10Panx early after SIV infection prevented HIV-associated synaptic damage in the white matter of the brain with long-lasting effects. Notably, they also found that administration of 10Panx early in infection reversed neuronal damage. Treatment with scrambled 10Panx did not result in the same inhibition of damage or maintenance of CD4+ and CD8+ cell count, affirming the potential for PANX1 inhibitors to be used in combination with ART (28).

Repurposing existing drugs with established safety profiles has gained popularity as a pragmatic near-term strategy to reduce cost and time to clinical translation compared with *de novo* drug development. Customarily prescribed for the treatment of gout, an autoinflammatory disease in which the accumulation of monosodium urate crystals in the synovial fluid of joints activates the NLRP3 inflammasome, probenecid was identified as a specific inhibitor of PANX1 and found to inhibit inflammasome activation in both astrocytes and neurons (28, 30–32). Able to permeate the BBB, probenecid has been explored as an adjuvant for tenofovir, an antiretroviral commonly prescribed to treat HIV (33). While the effects of long-term use of PANX1 inhibitors for that purpose are not well-understood, probenecid’s history of prolonged safe use for gout treatment suggests that the risk profile may be acceptable relative to potential benefits in selected high-risk populations.

Additional therapeutic approaches include the administration of oxidized ATP, a purinergic receptor antagonist, and enzymatic modulation of extracellular ATP via CD39 or CD73 (34–36). One challenge with these and other broad purinergic targeting approaches is their lack of specificity and potential off-target effects (37). Tissue penetration, BBB permeability, and maintaining a sufficient concentration in plasma are also limitations to the effectiveness of these approaches. It is these issues with tissue specificity and penetration that the introduction of nanobody-based inhibitors seeks to address (38). The nanobody HRB454 targets the same sequence as the mimetic peptide 10Panx, reducing inflammation in a similar manner without cytotoxicity (39). These approaches represent a promising direction for future therapeutic development, particularly in improving target specificity and tissue penetration.

### Purinergic receptor inhibition

Another possible approach to inhibiting the PANX1-ATP-P2X7 axis is to prevent engagement of purinergic receptors by extracellular ATP. General inhibition of purinergic receptors has been shown to inhibit the replication of both X4- and R5-tropic HIV (22, 40). A study of primary human macrophages found that, while P2X1, P2X7, and P2Y1 antagonists all inhibited viral replication, only the P2X1 antagonist NF279 prevented viral entry (24). P2X7 inhibition has also been shown to block the release of virions produced in infected macrophages (26). A previous study by our lab identified the necessity of purinergic signaling, particularly P2X receptors, for viral membrane fusion in both cell-free and cell-cell infection (40). Further, our lab identified the capacity of NF279 and NF449 to interact directly with HIV Env, possibly preventing binding of viral co-receptors CXCR4/CCR5 (41).

Purinergic inhibition through P2X antagonists, in addition to inhibiting infection, has been found to prevent the production of pro-inflammatory cytokines IL-10 and IL-1β in lymphoid tissue. While all antagonists were again highly effective at preventing productive infection, the P2X1-selective antagonist NF449 significantly lowered IL-10 and IL-1β release (42). These findings support purinergic inhibition as a strategy to limit HIV-associated inflammasome activation.

## Implications of inhibiting atp release

### Removal of an immune response pathway

Extracellular ATP functions as an early signal of cellular stress that activates purinergic receptor–mediated immune responses, raising the concern that its inhibition could impair protective immune responses, such as apoptosis. However, the NLRP3 inflammasome can be activated by multiple upstream signals (13), including danger-associated molecules, microbial products, and metabolic stressors (43–45). As such, inhibition of PANX1-mediated ATP release would not fully abrogate inflammasome activation but would selectively attenuate ATP-dependent signaling within this broader network.

Notably, PANX1 appears to play a more specific role in inflammasome activation during apoptosis, where ATP release contributes to NLRP3 signaling, while being dispensable in other activation contexts (46). Given that HIV-1 Env can induce apoptosis (47), selective inhibition of PANX1-mediated signaling may reduce inflammation and cellular damage while preserving other redundant innate immune pathways.

### Possibility of interfering with other pathways and cellular processes

When inhibiting part of a pathway like the PANX1-ATP-P2X7 axis, a key consideration is whether pannexin channel activity serves additional physiological roles that may be disrupted by its inhibition. PANX1 inhibition provides a unique opportunity in this regard, as PANX1 channels are typically low-activity and tightly regulated under physiologic conditions, so their inhibition would only affect inflammatory signaling (28).

PANX1-mediated ATP signaling has also been shown to impair the differentiation of CD34+ precursor cells, particularly in PWH who, despite ART, continue to have low CD4+ T cell counts. In these immune non-responders (INRs), P2X7 inhibition restored the T cell potential of their CD34+ hematopoietic progenitor cells, suggesting that inhibition of this pathway may not only reduce inflammation but also support immune reconstitution and reduce T cell exhaustion (48). This further supports the potential role of PANX1/P2X7 targeting as an adjuvant to ART, particularly in INRs.

### Timing of inhibition could affect effectiveness

HIV establishes a latent reservoir within days of primary infection, so any treatment aiming to prevent or reduce the establishment of a reservoir as a way of limiting inflammation must also begin during that period (49). Séror et al. suggests that the antiviral effect of PANX1 inhibition interrupts the HIV life cycle at the level of viral entry (22). One possible mechanism of this antiviral action is that PANX1 inhibition during acute infection prevents membrane depolarization, thereby preventing the formation of a viral synapse (22). Orellana et al. identified that HIV infection induces 2 separate PANX1 opening events, one within an hour of infection and another persistent opening 48–120 hours post- infection. By inhibiting PANX1 opening, they demonstrated a significant reduction in HIV replication in CD4+ T lymphocytes, a precursor cell to memory T cells that make up the HIV latent reservoir (23, 50). This result suggests that, if intervention occurs early in infection, PANX1 inhibition could restrict viral replication, thereby reducing reservoir size and later associated inflammation. Gorska et al. demonstrated the significance of early treatment on HIV-associated neuronal damage, showing that not only did early inhibition of PANX1-mediated ATP release reduce axonal/dendritic damage, but that early CNS damage caused by this pathway could be reversed (28).

For individuals with chronic HIV infection who have undergone long-term ART, PANX1 inhibition would not influence reservoir size, but inhibition of the pathway may still be effective for reducing persistent immune activation. PBMCs isolated from PWH have been shown to have spontaneously open PANX1, even in the absence of detectable viral replication (51). While the mechanism of this spontaneous opening is unknown, its existence suggests that, even long after acute infection, PANX1 inhibition can meaningfully reduce cellular damage and disease progression (25, 28).

## Beyond HIV

Both the immunopathogenic and proviral arms of the PANX1-ATP-P2X7 axis are present in other diseases. Rather, the widespread nature of this pathway suggests it may represent a conserved inflammatory signaling module across diseases. Comparative analysis of ATP-mediated signaling across disease contexts may further define key regulatory nodes within this pathway.

Recent studies have emphasized the effect of neuroinflammation on the severity of disease progression in Amyotrophic Lateral Sclerosis (ALS) (52, 53). IL-1β was identified as part of a inflammatory signature significantly associated with a higher risk of disease progression, and elevated NLRP3 inflammasome activation has been linked with neurodegeneration in individuals with ALS (53, 54). Similarly to HIV, it has been hypothesized that the upregulation of P2X7 receptors in spinal cord microglia and astrocyte and the corresponding ATP release could trigger the transformation of astroglia cells to a toxic phenotype that results in motor neuron damage (55). Although one is viral and the other is not, the involvement of the PANX1-ATP-P2X7 axis persists. These findings suggest that PANX1 inhibition may reverse neuronal damage beyond HIV-associated neuroinflammation.

HIV is not the only virus to modulate this ATP-mediated immune signaling to propagate viral spread. Epstein-Barr virus (EBV) an oncoherpesvirus, has been found to co-opt NLRP3 inflammasome assembly to induce viral reactivation. In patients with diabetes, inflammasome activation has been linked to the deregulation of EBV replication and a subsequent increase in risk for virus-associated lymphomas (56). In SARS-CoV-2 infection, the viral spike S1 protein induces sustained PANX1 opening, releasing extracellular ATP, which results in persistent downstream inflammasome activation and subsequent IL-1β release (57). The overlap in inflammatory signaling between these viruses potentially provides greater opportunities for treatment strategies.

The repurposing of probenecid from a gout medication to an inhibitor of viral infection illustrates the mutual benefits that arise from a better understanding of factors contributing to NLRP3 inflammasome activation. Medications that are effective inhibitors of inflammasome activation for other diseases could potentially be used for the treatment of HIV-associated inflammation and vice versa.

## Conclusion

Although antiretroviral therapy has progressed, the challenge of chronic inflammation caused by persistent HIV infection remains. Understanding the PANX1-ATP-P2X7 axis and resulting immune activation defines a mechanistic framework for understanding ATP-mediated immune communication in chronic inflammation. Inhibition of PANX1 and purinergic receptors represents a potential therapeutic strategy, but current options lack the specificity necessary. The development of therapeutic strategies to disrupt this inflammatory pathway extends beyond HIV, with potential relevance to the treatment of autoimmune diseases, other viral infections, and inflammatory conditions of the nervous system.

While this review is intended to present a conceptual framework, the prospect of translating this pathway into clinical practice highlights several key unanswered questions. These include identifying the patient populations most likely to benefit from PANX1–ATP–P2X7 targeting, such as individuals with persistent immune activation despite effective ART, and defining the safety and feasibility of long-term pathway inhibition. In addition, it remains unclear whether therapeutic modulation of this axis would be most effective as a continuous strategy or in a temporally restricted or context-dependent manner. Addressing these questions through mechanistic and clinical studies will be essential to determine whether targeting ATP-mediated immune communication can meaningfully improve outcomes for people with HIV and other chronic inflammatory conditions.

More broadly, the PANX1–ATP–P2X7 axis underscores the central role of extracellular nucleotide signaling in regulating immune communication across diverse disease states. As a conserved and context-responsive signaling mechanism, this pathway offers a framework for understanding how localized cellular stress can propagate into systemic inflammation. Future efforts should prioritize integrating mechanistic studies with biomarker-driven clinical investigation to determine whether targeted modulation of ATP-mediated signaling can be implemented safely and in a context-specific manner. In this way, insights derived from HIV may inform a broader strategy for identifying and therapeutically targeting dysregulated immune signaling networks in chronic inflammatory diseases.
